# Heat acclimation with probiotics-based ORS supplementation alleviates heat stroke-induced multiple organ dysfunction via improving intestinal thermotolerance and modulating gut microbiota in rats

**DOI:** 10.3389/fmicb.2024.1385333

**Published:** 2024-06-19

**Authors:** Lei Li, Juelin Chen, Yawei Wang, Yankun Pei, Lijun Ren, Xiaoyu Dai, Jinfeng Li, Jun Ma, Man Wang, Wenjun Chang, Jikuai Chen, Qing Song, Shuogui Xu

**Affiliations:** ^1^Department of Emergency, Changhai Hospital, Naval Medical University, Shanghai, China; ^2^Department of Emergency, The Second Naval Hospital of Southern Theater Command of PLA, Sanya, China; ^3^Heatstroke Treatment and Research Center of PLA, Sanya, China; ^4^Department of Health Toxicology, Faculty of Naval Medicine, Naval Medical University, Shanghai, China; ^5^Postgraduate School, Medical School of Chinese PLA General Hospital, Beijing, China; ^6^School of Traditional Chinese Medicine, Naval Medical University, Shanghai, China; ^7^Department of Rehabilitation, Changhai Hospital, Naval Medical University, Shanghai, China; ^8^Faculty of Naval Medicine, Naval Medical University, Shanghai, China

**Keywords:** heat acclimation, heat stroke, thermotolerance, probiotics, oral rehydration salt, gut microbiota, inflammation, multiple organ dysfunction

## Abstract

Heat stroke (HS) is a critical condition with extremely high mortality. Heat acclimation (HA) is widely recognized as the best measure to prevent and protect against HS. Preventive administration of oral rehydration salts III (ORSIII) and probiotics have been reported to sustain intestinal function in cases of HS. This study established a rat model of HA that was treated with probiotics-based ORS (ORSP) during consecutive 21-day HA training. The results showed that HA with ORSP could attenuate HS-induced hyperthermia by regulating thermoregulatory response. We also found that HA with ORSP could significantly alleviate HS-induced multiple organ injuries. The expression levels of a series of heat-shock proteins (HSPs), including HSP90, HSP70, HSP60, and HSP40, were significantly up-regulated from the HA training. The increases in intestinal fatty acid binding protein (I-FABP) and D-Lactate typically seen during HS were decreased through HA. The representative TJ proteins including ZO-1, E-cadherin, and JAM-1 were found to be significantly down-regulated by HS, but sustained following HA. The ultrastructure of TJ was examined by TEM, which confirmed its protective effect on the intestinal barrier protection following HA. We also demonstrated that HA raised the intestinal levels of beneficial bacteria *Lactobacillus* and lowered those of the harmful bacteria *Streptococcus* through 16S rRNA gene sequencing. These findings suggest that HA with ORSP was proven to improve intestinal thermotolerance and the levels of protective gut microbiota against HS.

## 1 Introduction

Heat stroke (HS) is a critical condition with extremely high mortality that is characterized by severe hyperthermia (> 40°C), central nervous system abnormalities (Liu et al., 2020). According to its etiological factors and susceptible population, HS is classified into classic HS and exertional HS. Owing to the recent trend of global warming, the increasing incidence of HS has become an increasing concern (Raymond et al., 2020). Although active on-site cooling and target temperature management in the early stages of HS can effectively alleviate its progression and reduce mortality, it is common for patients with HS to have difficulty obtaining effective cooling in real-world settings where the condition occurs often leading severe HS and death. It has been reported that the mortality rate for HS patients in intensive care units can exceed 60%.

After HS onset, it progresses rapidly and is often complicated by severe multiple organ injury if specific clinical treatment is not quickly initiated. Limited treatment options and a poor prognosis indicate that taking effective preventive measures is key to managing HS. Heat acclimation (HA) is widely recognized as the best and most economical measure to prevent and protect against HS, particularly for military personnel (Ashworth et al., 2020). HA refers to the improvement of thermal tolerance by exposure to hot environments and repeated heat stress stimulation. It is a routine strategy used to develop physiological adaptations against HS. Our group previously demonstrated that the protective effects of HA against HS are achieved in rats through upregulation of the expression of heat shock proteins (HSPs) expression.

In the pathophysiology of HS, the intestinal barrier plays a significant role in defending against lipopolysaccharides (LPSs), endotoxins, and other toxic substances. It has been reported that intestinal barrier injury can occur during the early stages of HS, and that this barrier is sensitive to heat stress (Tang et al., 2023). Furthermore, HA has been found to alleviate intestinal lesions in mice with HS (Huang et al., 2023). Therefore, maintaining the intestinal barrier’s integrity and improving its thermotolerance during heat stress might help prevent the onset and subsequent pathology of HS. Some studies have found that the preventive administration of oral rehydration salts III (ORSIII) can protect intestinal function in rats with HS, and improve body thermotolerance during exercise in the heat. A previous study by our group found that the preventive administration of probiotics could also improve intestinal barrier function against HS by regulating gut microbiota. Based on these findings, we further hypothesized that HA with probiotics-based ORS (ORSP) supplementation might alleviate HS-induced multiple organ injury by improving intestinal thermotolerance and microbiota.

In this study, we established a rat model of HA with ORSP, during a consecutive 21-day HA training regimen. We then assessed the effects of HA with ORSP against HS by detecting the degree of multiple organ injury. Finally, we evaluated the intestinal thermotolerance and microbiota of the rats, to clarify the mechanism underlying HA with ORSP.

## 2 Materials and methods

### 2.1 Animals

**Figure 1:**
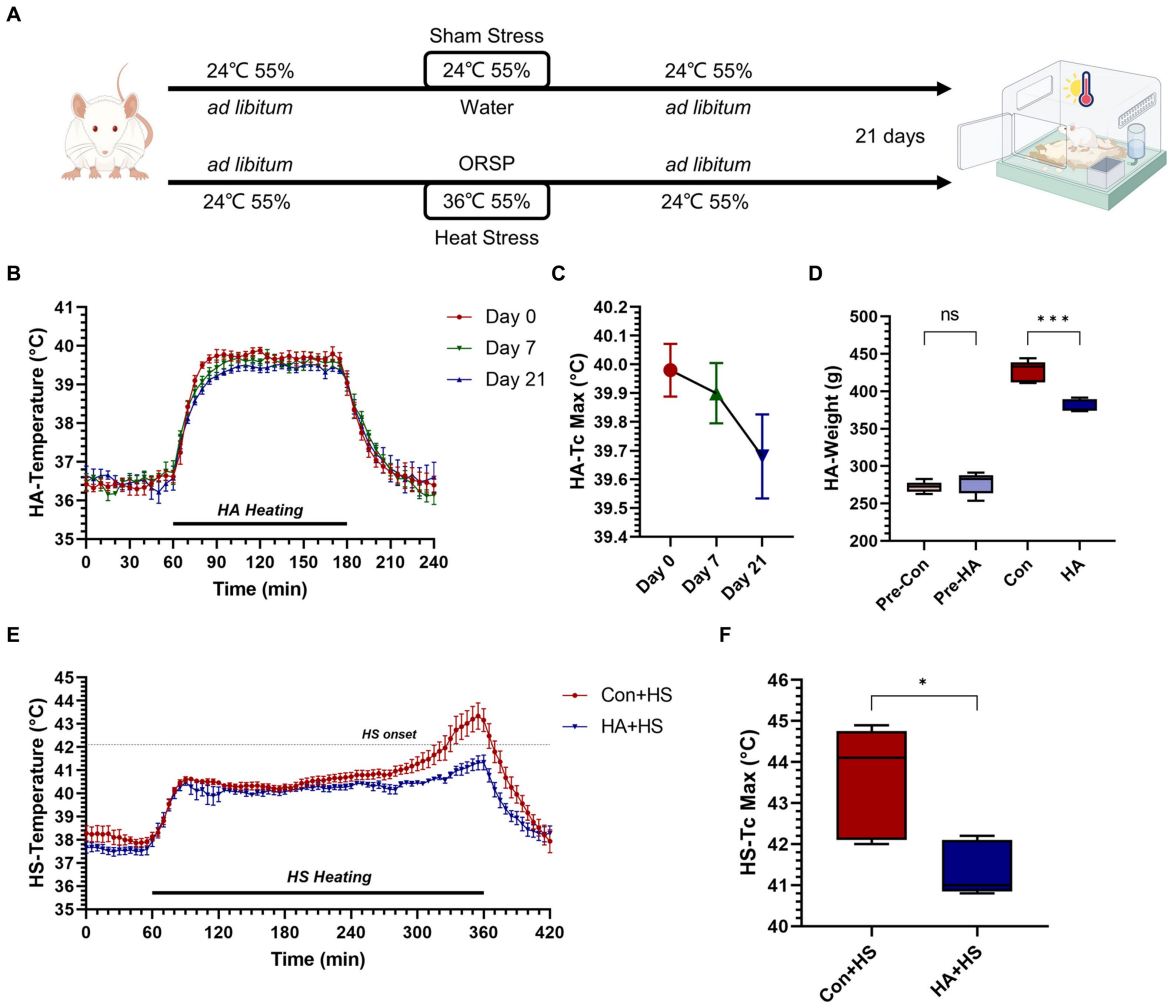
Heat acclimation protocol and thermoregulatory response characteristics in HA and HS. **(A)** Rats were exposed to heat stress at 36°C and 55% relative humidity for 2 h per day for 21 days with probiotics-based ORS supplementation (HA group). **(B)** Core body temperature (Tc, °C) was continuously monitored during HA and Tc curves on day 0, 7, and 21 were compared. **(C)** The maximum Tc (Tc Max) during heat acclimation on day 0, 7, and 21 were compared. **(D)** Body weight changes on day 0 and 21were compared. **(E)** Tc curves during heat stroke were monitored and compared between HS groups. **(F)** Tc Max during HS were compared. Data in panel **(B,C,E)** are presented as the mean ± SEM of 5 rats per group. Data in panel **(D,F)** are presented as a box-and-whisker plot of 5 rats per group. ns *p* > 0.05, ^∗^*p* < 0.05, ^∗∗^*p* < 0.01, ^∗∗∗^*p* < 0.001.

Male Sprague–Dawley rats (7–8 weeks old, 250–300 g), were purchased from Sippr B&K Laboratory Animal Ltd. (Shanghai, China). All rats were raised in the specific pathogen free (SPF) Animal Experiment Center of the Naval Medical University in China and acclimated to a room with a 22 ± 1°C ambient temperature, 50 ± 5%relative humidity (RH), and 12-h day/night cycle for 1 week prior to the study. All experimental procedures were approved by the Institutional Animal Ethics Committee of the Naval Medical University (reference number: NMUM-REC-2021-002) according to the Guide for the Care and Use of Laboratory Animals of the National Institutes of Health. A total of 44 rats were used in this study. Ten rats were used to obtain core body temperature (Tc) characteristics under heat stress, 10 rats were used to obtain fecal data to conduct gut microbiota examinations, and 24 rats were used to obtain blood and organ samples for further study. As is shown in Figure 1A, the rats were randomly allocated into two groups according to whether they underwent HA training. After HA training, all rats that met the experimental goals were then divided into four groups according to whether they underwent HS induction. Therefore, four distinct groups of rats were used in this study. The control group rats (Con, *n* = 16) received neither HA nor HS treatment. The HA group rats (HA, *n* = 16) were only subjected to HA. The Con + HS group rats (*n* = 6) were only subjected to HS. The HA + HS group rats (*n* = 6) were subjected to both HA and HS. All the rats in this study were anesthetized using isoflurane. Any rat that failed to achieve the experimental objectives during the course of the experiment were euthanized if they were unable to live normally (e.g., owing to severe pain and discomfort caused by the experimental procedures). All euthanasia methods used in our lab were performed using specialized euthanasia instruments (KW-AL; Calvin Bio Technology Co., Ltd., Nanjing, China) for carbon dioxide inhalation. All experimental animals were monitored for vital signs for >5 min following euthanasia, to confirm death.

### 2.2 Heat acclimation and heat stroke protocols

The HA protocol was conducted according to previously reported protocols used by our group, as well as recent advances in HA study (Cao et al., 2022; Huang et al., 2023; Li et al., 2023). Briefly, rats in the HA group were placed in an artificial climate chamber (LTH-575 N-01; Shanghai Drawell Scientific Instrument Co., Ltd., Shanghai, China) where environmental parameters were maintained at 36 ± 1°C and 55 ± 5% RH for 2 h per day, for 21 days. The drinking water for the rats undergoing HA training was supplemented with ORSP (Beijing OASIS Technology Co., LTD, Beijing, China). The ORSP used in this experiment was composed of ORSIII (each package of ORSP contains 0.65 g of sodium chloride, 0.725 g of sodium citrate, 0.375 g of potassium chloride, and 3.375 g of anhydrous glucose, which is consistent with the composition of regular ORS III formula) and three additional of probiotics (*Lactobacillus acidophilus NCFM*, *Lactobacillus rhamnosus LGG*, and *Bifidobacterium lactis HN019*). The configured ORSP had the same osmolality as the standard ORSIII. During HA training, ORSP combined with standard pellet rat feed was provided *ad libitum*. Accordingly, the control rats were provided with standard pellet rat feed and distilled tap water *ad libitum*. As is shown in Figure 1A, the rats who were subjected to HA training for 21 days without injury were used for the subsequent experiments.

The HS protocol was conducted according to our previously reported protocol, with some minor improvements. An artificial climate chamber where the ambient temperature was maintained at 40 ± 1°C and RH at 60 ± 5%, was used to induce HS onset. We replaced closed plastic cages with hollowed-out metal cages to ensure absolute uniformity of the ambient temperature and RH to avoid overcrowding leading to additional heat production. The timepoint of stable hyperthermia occurrence (core body temperature [Tc] > 42°C) was taken as the main standard indicating HS onset in the Con + HS rats. To ensure that the intensity of the heat stress received was consistent, the experimental rats in the HA + HS group were removed at the same time. All of the rats with successful HS induction were then transferred to the regular environment with *ad libitum* access to food and water. At 3 h after HS induction, various samples such as blood serum, plasma, organ tissues, and fecal matter were gathered, using a sample collection protocol described in our previous study (Li et al., 2021). The temperature-monitoring capsules (SV223 capsule thermometer, Shenzhen Flamingo Technology Co., Ltd.) were implanted 3 days before the start of the animal experiment in the experimental rats to obtain Tc readings at 5-min intervals.

### 2.3 Serum biochemical markers and inflammatory cytokine analysis

The rats’ levels of alanine aminotransferase (ALT), aspartate aminotransferase (AST), blood urea (BU), creatine kinase (CK), and creatinine (CREA) were analyzed using an automated biochemical analyzer (7,080 automated analyzer, HITACHI, Tokyo, Japan). Plasma D-Lactate and intestinal fatty acid-binding protein (I-FABP) were, respectively, detected by a D-Lactate Colorimetric Assay Kit (BioVision, Milpitas, CA, USA) and a Rat I-FABP enzyme-linked immunosorbent assay kit (Sangon Biotech, Shanghai, China) according to the manufacturers’ instructions.

### 2.4 Western blot assay

Frozen intestinal tissues (100 mg) from each group were ground into powders in liquid nitrogen, lysed on ice using radioimmunoprecipitation assay (RIPA) buffer (Beyotime Institute of Biotechnology, China) containing protease and phosphatase inhibitor cocktail (Roche Diagnostics GmbH, Germany), and centrifuged at 12,000 rpm for 30 min at 4°C. Subsequently, the supernatant was and the protein concentration was measured using a bicinchoninic acid (BCA) protein assay kit (Beyotime Institute of Biotechnology, China), before detecting the protein expression by western blot. Protein samples were mixed with loading buffer, denatured, and separated on 4–12% and 4–20% SurePAGE™ gels (GenScript, Nanjing, China). Protein electrophoresis membranes were transferred using an eBlot™ L1 Fast Wet Transfer System (GenScript, Nanjing, China). The blotted wet membrane was blocked for 2 h at room temperature with blocking buffer (Beyotime Institute of Biotechnology, China). Next, the membranes were incubated overnight with primary antibodies at 4°C, followed by incubation with secondary antibodies for 2 h at 37°C. The Omni-ECLTM Femto Light Chemiluminescence Kit was used to detect luminescence signals on the membrane, and the Amersham Imager 600 (GE Healthcare Bio-Sciences AB) was used to image the blots. The following primary antibodies were used overnight at 4°C: HSP90 (proteintech, 13171-1-AP, 1: 4000, rabbit), HSP60 (proteintech, 15282-1-AP, 1:4000, rabbit), HSP40 (proteintech, 13174-1-AP, 1:5000, rabbit), HSP70 (proteintech, 10995-1-AP, 1:10000. rabbit), Junctional Adhesion Molecule 1 (JAM-1) (Beyotime, AF2146, 1:1000, rabbit), Zonula Occludens-1 (ZO-1) (proteintech, 21773-1-AP, 1:5000, rabbit). E-cadherin (proteintech, 20874-1-AP, 1:40000, rabbit), and GAPDH (Beyotime, AF0006, 1:1000, mouse).

### 2.5 Histological examination and transmission electron microscopy

All of the organ tissues were treated according to our previously described protocol (Li et al., 2021; Li L et al., 2024). Briefly, the organ samples were fixed, dehydrated, embedded, sectioned and stained with Hematoxylin and Eosin (H&E). All the H&E-stained slides were examined using a light microscope (DM2000, Leica, Wetzlar, Germany) and scanned using a panoramic MIDI slide scanner (3DHISTECH, Hungary). Similarly, colon samples for transmission electron microscopy (TEM) examination were harvested and fixed with 1% OsO4 in 0.1 M phosphate-buffered saline (PBS; pH 7.4) for 2 h at room temperature. The fixed samples were then dehydrated, embedded, and polymerized. Ultrathin sections (60–80 nm) were cut and stained with 2% uranyl acetate and 2.6% lead citrate. The sections were observed, and images were scanned under a Hitachi-7000 electron microscope (Hitachi, Naka, Japan).

### 2.6 Gut microbiota 16S rRNA sequencing and analysis

All fecal samples were treated according to the protocol described in our previous study (Li et al., 2021). DNA was extracted from soil samples using an E.Z.N.A.® soil DNA kit (Omega Bio-tek, in Norcross, GA, USA). Subsequent polymerase chain reaction amplification was performed using an AxyPrep DNA gel extraction kit (Axygen Biosciences, in Union City, CA, USA). Finally, Illumina MiSeq sequencing was performed on an Illumina MiSeq PE300 platform (Illumina, San Diego, CA, USA), according to the manufacturer’s instructions. The unprocessed 16S rRNA gene sequencing data were analyzed using the Quantitative Insights Into Microbial Ecology (QIIME) platform with i-Sanger platform4 from Majorbio BioTech Co., Ltd. (Shanghai, China). The unprocessed data has been submitted to the Sequence Read Archive (SRA) database under accession number PRJNA1074273.Alpha diversity indexes were used to assess community diversity briefly. An examination of variations in beta-diversity via principal coordinate analysis (PCoA) was carried out at the OTU level using weighted UniFrac distances. ANOSIM/Adonis was used to assess variances among groups. Wilcoxon rank-sum or Mann–Whitney U tests were used to compare pairs of groups at the phylum and genus levels. Research was conducted to examine how GS impacted the microbiota in the gastrointestinal tract. Bar graphs displaying species composition analyses were created using the results of taxonomic and statistical analysis. The LEfSe algorithm, which combines LDA with effect size measurements, was used to identify taxa that distinguish identified taxa between two metadata classes, as well as determine any significantly abundant and biologically relevant features in the groups.

### 2.7 Statistical analysis

Experimental results are displayed as mean values with standard errors. GraphPad Prism version 9.4.0 (GraphPad Software, La Jolla, CA, USA) was used for all statistical analyses excluding the microbiota data, which were analyzed using multivariate and advanced statistical methods, according to a previously described approach (Li et al., 2021). Data from various groups were examined via one-way analysis of variance, followed by the Tukey–Kramer multiple comparison test. The two-tailed Student’s t-test was used to compare results from two independent groups. A bidirectional *p*-value of ≤0.05 was taken to indicate statistical significance.

## 3 Results

### 3.1 Heat acclimation with ORSP improved thermoregulatory response during heat stress

The rats were continuously exposed to heat stress for 21 days with ORSP supplementation to achieve HA, as is shown in Figure 1A. Over 21 days of HA, Tc was continuously monitored during training and Tc curves were compared on day 0, 7, and 21 (Figures 1B,C). The maximum Tc (Tc Max) decreased continuously during HA training (day 0, 40.0°C ± 0.205; day 7, 39.9°C ± 0.235; day 21, 39.7°C ± 0.327). Furthermore, HA induced weight loss vs. the Con group but had no effect on health (Figure 1D; Table 1). HS induction was then performed in the rats that completed HA training, and their Tc curves were monitored (Figure 1E). Importantly, the rats in HA + HS group had lower Tc in the compensatory phase of the thermoregulatory response during HS induction. However, the rats in the Con + HS group rapidly progressed to hyperthermia in the last decompensated phase of the thermoregulatory response during HS induction, while those in the HA + HS group only experienced mild hyperthermia. Further comparing the Tc Max values of the rats during HS induction (Figure 1F), we saw that the Tc Max values of the rat in the HA + HS group were significantly lower than those of the Con + HS group rats (Table 1, 41.4°C ± 0.665 vs. 43.6°C ± 1.36; *p* < 0.01; n = 5 per group).

**Table 1:** Experimental records and graphic characteristics of rats subjected to HA and HS.

	Con	HA	Con + HS	HA + HS
BW pre-gavage, g	272.00 ± 7.38	277.02 ± 14.50	–	–
BW post-gavage, g	426.92 ± 14.40	380.56 ± 8.41^***^	–	–
Tc Max, °C	–	–	43.56 ± 1.36	41.38 ± 0.66^#^
ALT, U/L	45.88 ± 6.49	44.23 ± 5.64	202.71 ± 84.47^&&&^	68.78 ± 20.10^###^
AST, U/L	147.95 ± 49.10	151.76 ± 17.13	1091.52 ± 433.73^&&&^	361.98 ± 103.58^###^
BU, mmol/L	5.75 ± 0.68	8.15 ± 1.13	19.40 ± 3.46^&&&^	12.03 ± 3.00^###^
CK, U/L	847.08 ± 458.7	677.65 ± 69.85	3048.80 ± 1313.26^&&&^	1438.98 ± 447.16^##^
CREA, μmol/L	50.33 ± 5.71	63.23 ± 6.72	85.72 ± 17.65^&&&^	74.03 ± 17.46
I-FABP, ng/mL	7.14 ± 1.21	7.30 ± 1.02	18.43 ± 4.12^&&&^	14.47 ± 0.98^#^
D-Lactate, μmol/mL	1.53 ± 0.25	1.42 ± 0.33	3.20 ± 0.42^&&&^	2.62 ± 0.30^###^

Data are shown as means ± SD; BW, body weight, Tc Max, mean value and SD of the maximum Tc during the heat stroke period. ALT, alanine aminotransferase; AST, aspartate aminotransferase; BU, blood urea; CK, creatine kinase; CREA, creatinine; I-FABP, intestinal fatty acid binding protein; ^#^*p* < 0.05, ^##^*p* < 0.01, ^∗∗∗,&&&,###^*p* < 0.001.

### 3.2 Heat acclimation with ORSP alleviated HS-induced multiple organ injury

**Figure 2:**
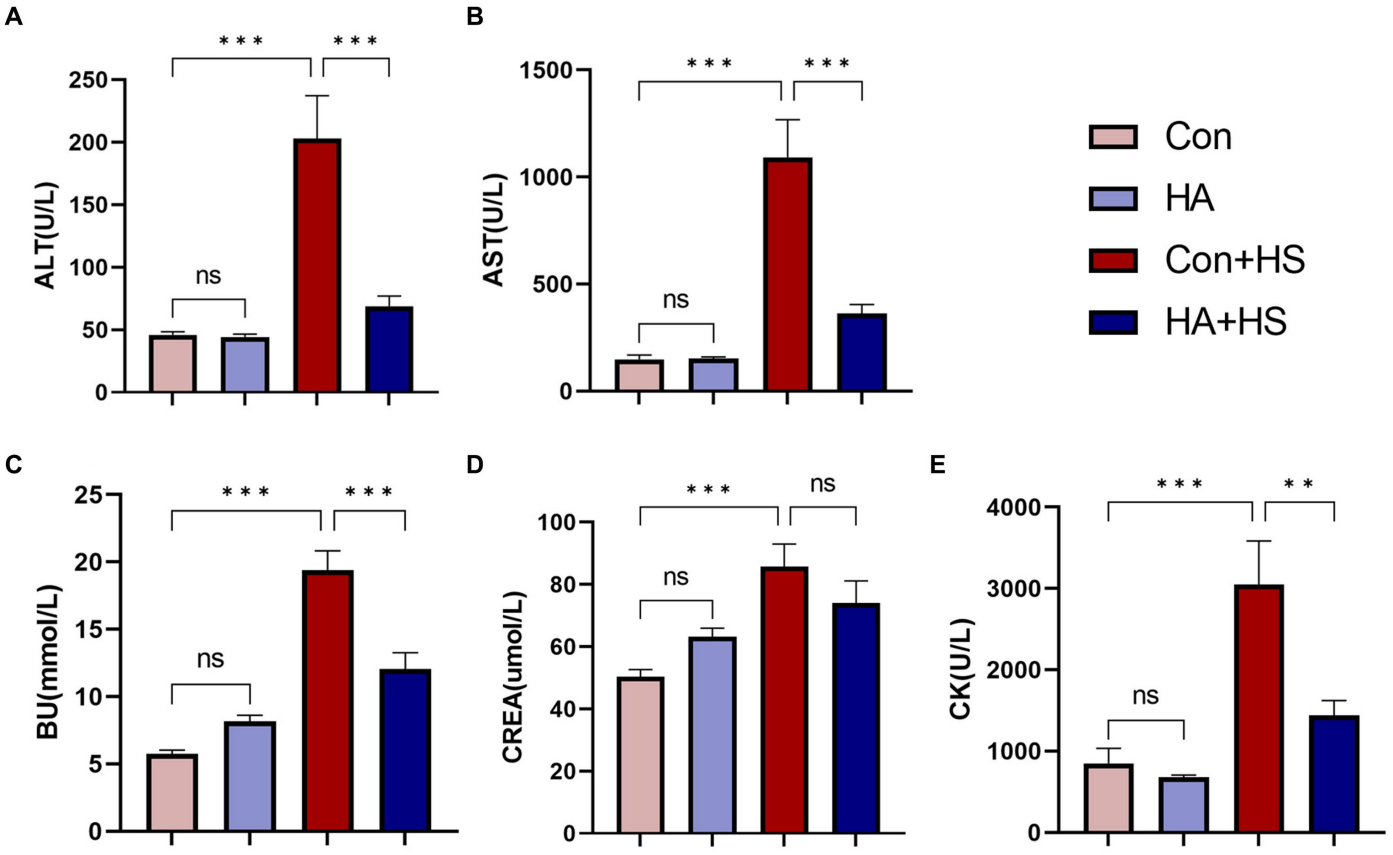
HA with ORS decreased levels of serum biochemical markers in rats. Blood samples of rats in each group were collected 3 h after HS induction. Blood samples of rats in each group were collected 3 h after HS induction. Serum biochemical indices of ALT **(A)**, AST **(B)**, BU **(C)**, CREA **(E)**, and CK **(D)** were examined and compared between groups. Data are presented as means ± SEM, n = 6 per group. ns *p* > 0.05, ^∗^*p* < 0.05, ^∗∗^*p* < 0.01, ^∗∗∗^*p* < 0.001. ALT, alanine aminotransferase; AST, aspartate aminotransferase; BU, blood urea; CK, creatine kinase; CREA, creatinine.

**Figure 3:**
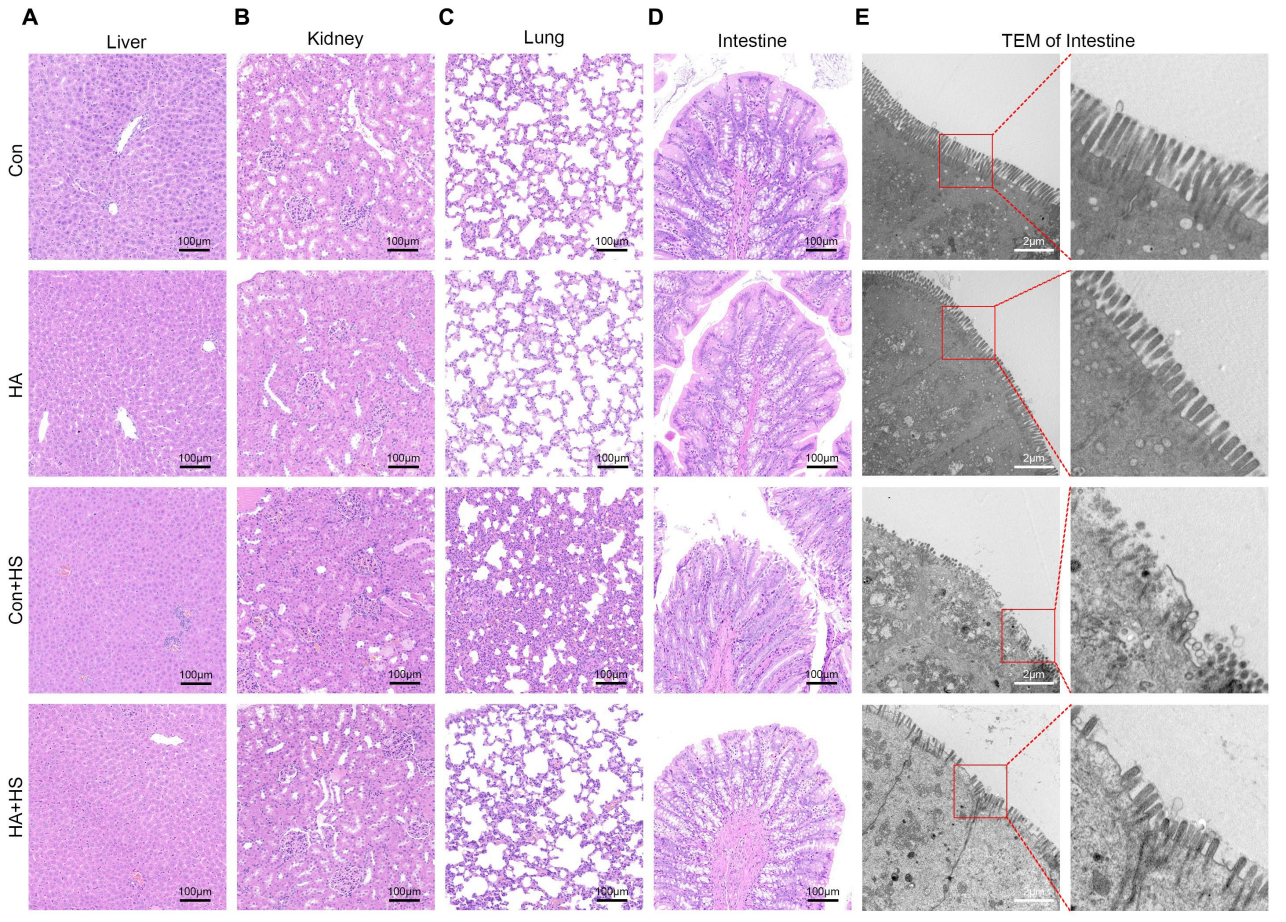
HA with ORSP alleviated multiple organ injury in rats. Organ samples of rats in each group were harvested at 3 h after HS induction and stained with H&E. Representative H&E images are shown if liver **(A)**, kidney **(B)**, lung **(C)**, and intestine **(D)** from each group. Magnification 100 ×. Scale bar = 100 μm. Typical pathological changes including necrosis, inflammation, vacuolar degeneration, congestion, hemorrhage, and alveolar wall thickening were observed in Con + HS group and were found to be attenuated in HA + HS group. **(E)** Representative ultrastructure transmission electron photomicrographs of intestine in each group show the intestinal barrier morphology and structure. Typical sites framed in red are magnified and shown. Scare bar = 2.0 μm, magnification 8,000 × .

Blood and organ samples from rats in each group were harvested at 3 h after HS induction and stained with HE. As is shown in Figure 2 and Table 1, the serum biochemical indexes related to ALT, AST, BU, CREA, and CK were found to be significantly increased in the Con + HS group, which indicated that multiple organ injury developed during HS. Typical features of tissue injury such as edema, inflammation, congestion and necrosis were also observed in those organs of the Con + HS group rats (Figures 3A–D). However, HA with ORSP inhibited the increase of serum biochemical indexes and alleviated HS-induced multiple organ injury to a significant degree, which indicated that HA training was able to reduce the risk of HS onset in rats.

### 3.3 Heat acclimation with ORSP improved intestinal thermotolerance

**Figure 4:**
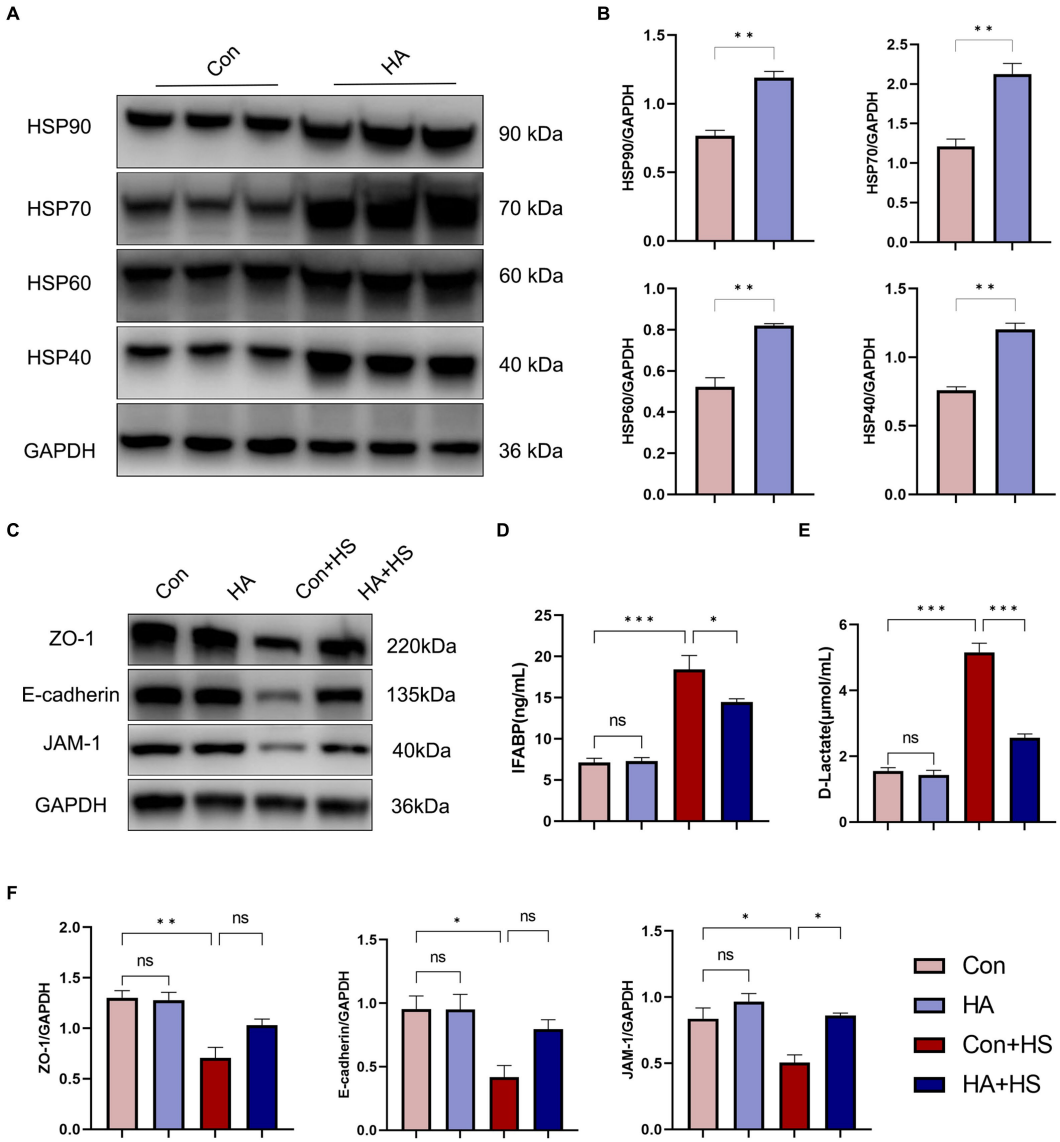
HA with ORSP improved intestinal thermotolerance. **(A)** The expression of HSPs in intestine was detected by western blot. GAPDH was used as a loading control to conduct quantitative analysis between Con and HA groups **(B)**. **(C)** The expression of TJs in intestine was detected and quantitatively compared **(F)** among four groups. Plasma I-FABP **(D)** and D-Lactate **(E)** were detected at 3 h after HS induction. Data are presented as means ± SEM, **(B)** *n* = 3 per group, **(D,E)** *n* = 6 per group. ns *p* > 0.05, ^∗^*p* < 0.05, ^∗∗^*p* < 0.01, ^∗∗∗^*p* < 0.001.

The expression levels of HSPs in the freshly isolated intestinal samples of the rats subjected to HA training was assessed via western blot and compared with those of the unacclimated control rats (Figure 4A). Protein band densities were normalized to GAPDH and quantified for comparison (Figure 4B). The expression levels of several HSPs, including HSP90, HSP70, HSP60, and HSP40, were found to be significantly up-regulated after HA training. Levels of the classical biomarkers I-FABP and D-Lactate were also detected, to evaluate the degree of intestinal injury. The levels of I-FABP and D-Lactate were found to be significantly higher in Con + HS group compared to the HA + HS one (Figures 4D,E; Table 1). The representative TJ proteins including ZO-1, E-cadherin, and JAM-1 were found to be markedly down-regulated during HS and sustained in the rats that had undergone HA (Figures 4C,F). We also examined the ultrastructure of TJ via TEM (Figure 3E). In the Con and HA groups, normal cell structures, mitochondria, aligned microvilli, and intact TJ structures were initially observed—all of which were severely disrupted by HS. In particular, the widespread damaged microvilli and discrete TJs indicated that the integrity of the intestinal barrier was damaged. Consistent with previous results, the ultra-structures of the TJs were found to be partially maintained in the HA + HS group. Altogether, the results indicated that HA with ORSP improved intestinal thermotolerance against HS-induced intestinal barrier disruption in our rat model.

### 3.4 Heat acclimation with ORSP modulated the structure and composition of gut microbiota

**Figure 5:**
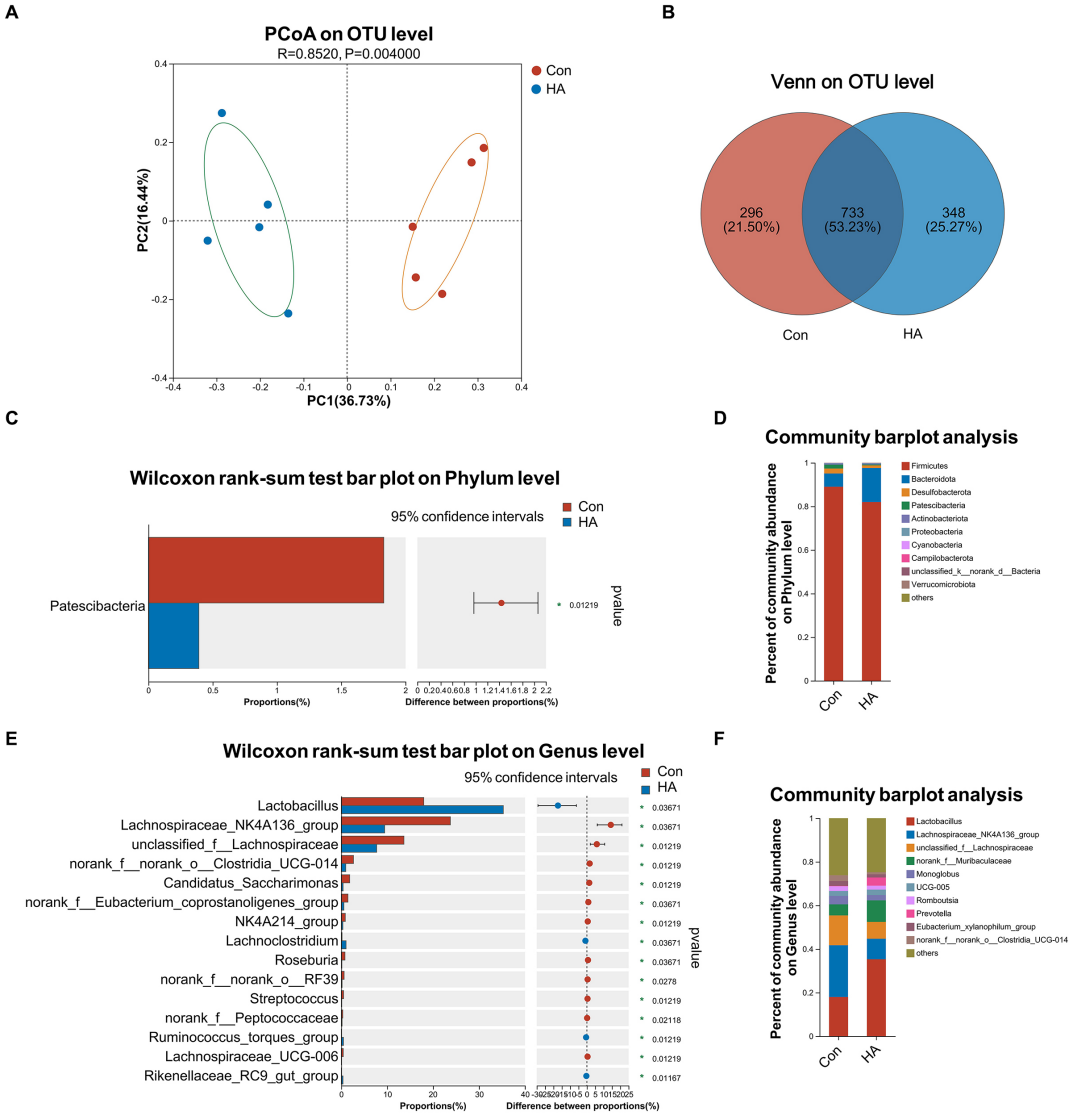
HA with ORSP modulated the overall structure of gut microbiota of rats. **(A)** Principal coordinate analysis (PCoA) was conducted to determine difference between two groups. **(B)** Venn diagram on OTU level showed shared and unique OTUs between two groups. **(C, D)** Average relative abundances of microbial community composition for two groups was shown by bar plots for the phylum level, and 1 phylum was found with significant difference. **(E, F)** Average relative abundances of microbial community composition for two groups was shown by bar plots for the genus level, and the top 15 abundant genera with significant difference are shown. Data are shown as the mean by bar plot analysis. *n* = 5 per group. *p < 0.05.

The gut microbiota of our various rat groups was then investigated via 16S rRNA gene sequencing. PCoA analysis demonstrated that the HA group differed significantly from the Con group at the OTU level, based on Bray-Curtis analysis (Figure 5A; R = 0.852, *p = 0.004*). A Venn diagram at the OTU level indicated that the two groups shared 733 (53.23%) identical OTUs, and that each one had 296 (21.50%) and 348 (25.27%) unique OTUs, respectively (Figure 5B). Their Shannon and Chao diversity, and Ace and Sobs microbial community indexes were compared at the OTU level (Supplementary Figures 1A–E). Their gut microbiota compositions were assessed via bar plot analysis, as is shown in Figures 5D,F. At the phylum level, only one phylum (*Patescibacteria*) was identified to differ significantly between the two groups by the Wilcoxon rank-sum test (Figure 5C). At the genus level, the top 15 abundant genera with significant differences are shown in Figure 5E. Among these genera, *Lactobacillus*, *Lachnoclostridium*, *Ruminococcus torques,* and *Rikenellaceae RC9* were found to be significantly upregulated in the HA group. Conversely, *Lachnospiraceae NK4A136*, *Roseburia*, *Streptococcus*, and eight other genera were found to be significantly downregulated in the HA group. The results of our LEfSe analysis (LDA > 2.0, *p* < *0.05*) were consistent with the pairwise comparisons detailed above (Supplementary Figure 1G). *Lachnospiraceae NK4A136* was the most significant bacterial family identified in the Con + HS group, while *Lactobacillus* was the most significant one in the HA + HS one.

## 4 Discussion

HS has been attracting increasing attention worldwide, in recent years. As previously described in the expert consensus published by our team (Liu et al., 2020), HS is a fatal condition with a very high mortality rate. The pathogenesis of HS involves a transition from a compensable thermoregulatory state to a non-compensable one. Thermoregulatory failure exacerbates the pathophysiological processes involved—including severe inflammation, multiple organ injury, and disseminated intravascular coagulation—which result from the combination of extreme hyperthermia and circulatory collapse (Epstein and Yanovich, 2019; Li R et al., 2024). Prompt recognition and effective on-site cooling can rapidly reverse the organ dysfunction caused by HS, in most cases. However, cooling treatment constrained by limited resource availability may not suffice to achieve full recovery. Once patients with HS develop systemic inflammation responses and multiple organ failure, even intensive treatments may not be sufficient to prevent death. Because HS can easily become severe once it occurs, and its prognosis is often poor even after intensive treatment in hospital, it is particularly important to develop early preventive measures to prevent its onset. It has been reported that HA training can improve thermotolerance and reduce the risk of HS onset (Ashworth et al., 2020). Thermotolerance achieved through HA is characterized by reversible phenotypic changes (Murray et al., 2022). Our previous study successfully demonstrated that HA could attenuate HS-induced organ injury by upregulating the expression of HSPs (particularly HSP70 and HSP90). We also found that supplementation with probiotics for 7 days could significantly modulate gut microbiota and sustain the intestinal barrier, thereby preventing HS onset. It has also been reported that pretreatment with ORSIII can prevent the occurrence of HS-induced intestinal lesions, as evidenced by several serum biochemical indexes and I-FBAP (Lin et al., 2019). One study demonstrated that HS could lead to kidney injury, but that ORSIII supplementation could alleviate this injury by assessing Cr, BU, and NGAL. A team found that ORS intake during exercise in heated conditions could decrease muscle cramp susceptibility (Lau et al., 2021). Moreover, another team (Schleh and Dumke, 2018) found that ORS intake during outdoor work was effective for preventing industrial accidents and HS in hotter environments, which suggests that it is important to select appropriate drinks with sufficient electrolytes when working under such conditions. Encouragingly, Huang et al. found that HA could induce changes in gut microbiota, thereby reducing inflammation by inhibiting NF-κB signaling and increasing TJ protein expression (Huang et al., 2023). Thus, the combination of ORS and probiotics during HA regimens may help accelerate improvements in thermotolerance and protect against HS. Notably, we found that the combination of ORS and probiotics has been previously investigated for treating diarrhea in children (Ardakani et al., 2022; Kodam, 2022). An ORSP formulation containing three kinds of edible probiotics for infants (*Lactobacillus acidophilus NCFM*, *Lactobacillus rhamnosus LGG*, and *Bifidobacterium lactis HN019*) that has been certified by the National Health Commission of China was selected for this experiment.

In this study, we first examined the effect of HA on the thermoregulatory response. As expected, we found that Tc Max decreased in the HA-trained rats, by day 21. We then continued to monitor their Tc values during HS induction. We also observed that HA could reduce the body’s thermoregulatory response and delay the occurrence and severity of hyperthermia in HS, which is consistent with the findings of previous reports. Blood biochemical tests and HE histological observations further confirmed that HA could alleviate HS-induced multiple organ injury. Thus, we confirmed the preventive effects of HA with ORSP with regard to HS. Intestinal injury plays a key role in the pathophysiology of HS, and previous studies have shown that HA can prevent the intestinal injury induced by HS. Our subsequent work has therefore focused more specifically on the involvement of the intestines. The elevated expression levels of several HSPs that we found in our HA-trained rats demonstrated that HA training may improve intestinal thermotolerance by regulating the heat shock response. In fact, upregulation of the heat shock response by HA, via promoting increased HSP expression, has been shown to protect cells from heat stress damage (Nava and Zuhl, 2020). Our results proved that HA with ORSP could sustain intestinal barrier integrity during HS—as evidenced by TJ protein expression, intestinal injury biomarkers, and TEM images. Overall, these results demonstrate that HA can enhance intestinal tolerance to heat stress injury (i.e., thermotolerance).

Considering that both HA and probiotics have positive effects on gut microbiota, we also performed 16 S rRNA sequencing in our experimental rats. Our PCoA and Venn analyses indicated that HA with ORSP could significantly alter gut microbiota compositions. We further investigated microbial community structure changes and detected levels of specific bacterial genera. *Lactobacillus*, *Lachnoclostridium*, *Ruminococcus torques,* and *Rikenellaceae RC9* were found to be present in significantly higher levels in our HA group. It is worth noting that *Lactobacillus* represents one of the components of the ORSP formula we used, as well as a key probiotic reported to benefit HA in several other reports (Cao et al., 2022; Huang et al., 2023). This suggests that our HA protocol was scientifically effective, and that supplementation with ORSP provided a positive beneficial effect. *Lachnospiraceae NK4A136*, *Roseburia*, *Streptococcus*, and eight other genera were found to be significantly downregulated in our HA group. Among these genera, *Streptococcus* is generally considered a harmful bacterium that is associated with various gastrointestinal disorders such as irritable bowel syndrome, inflammatory bowel disease, and Crohn’s disease (Quaglio et al., 2022; Wang et al., 2022). We found that HA with ORSP was able to upregulate the abundance of probiotic bacteria and downregulate that of harmful ones. We also demonstrated that HA with ORSP was able to improve the gut microbiota structures of rats.

We thus concluded that HA with ORSP further enhanced the effect of HA with regard to improving thermotolerance. This improved thermotolerance was mainly reflected in the upregulation of the intestinal heat shock response and the stability of tight junction structures. The intestines, as the central starting organ of the HS pathogenesis, gain increased resilience through HA with ORSP, thus playing a pivotal role in defending against lipopolysaccharide, endotoxin, and other toxic substance infiltration during HS. However, the relationship between the heat shock response, intestinal barrier function, and gut microbiota nevertheless merits further investigation—particularly in terms of HA and HS. It is also worth to note that both HA and HS are involved in the problem of rhabdomyolysis, and whether ORSP supplementation can help solve rhabdomyolysis is also worth verifying in follow-up studies (He et al., 2020, 2022; Li et al., 2022). It has been reported that a kind of probiotic *Akkermansia muciniphila* could improve heat stress-impaired intestinal barrier function by modulating HSP27, which suggests that it is important to study the relationship between probiotics and HSPs. Recently, MLCK/MLC2 (Du et al., 2023), TLR3/TRIF/RIP3 (Zhou et al., 2023), TLR4/STAT6/MYLK (Niu et al., 2023), ROS/JNK (Pan et al., 2022), and p-eif2α/CHOP (Cui et al., 2022) pathways were found to be associated with heat stress related intestinal barrier dysfunction. But in the field of HA training, intestinal injury related pathways mechanism research is relatively scarce. A study found that HA could alleviated HS-induced intestinal lesion by inhibiting NF-κB signaling pathway. We hypothesized that the combined treatment of HA and ORSP may play a protective role against HS by inhibiting the inflammatory response and gut barrier damage pathways through regulating intestinal flora and heat shock protein expression. In the future, we will explore and verify the corresponding mechanisms from these perspectives.

## 5 Conclusion

This study indicated that HA with ORSP could effectively improve intestinal thermotolerance and gut microbiota compositions in rats, through consecutive 21-day heat stress exposure. Furthermore, HA with ORSP significantly alleviated HS-induced multiple organ injury. We also confirmed that HA could upregulate the beneficial intestinal bacteria *Lactobacillus*, and downregulate harmful bacteria *Streptococcus*. In the future, the relationship between the heat shock response, intestinal barrier function, and gut microbiota should be explored to fully clarify the mechanism behind HA and inform the development of more efficient HA training protocols.

## Data availability statement

The datasets presented in this study can be found in online repositories. The names of the repository/repositories and accession number(s) can be found in the article/Supplementary material.

## Ethics statement

The animal study was approved by the Institutional Animal Ethics Committee of the Naval Medical University. The study was conducted in accordance with the local legislation and institutional requirements.

## Author contributions

LL: Data curation, Investigation, Writing – original draft, Writing – review & editing. JuC: Investigation, Writing – original draft. YW: Investigation, Writing – original draft. YP: Investigation, Writing – original draft. LR: Investigation, Writing – original draft. XD: Investigation, Writing – original draft. JL: Investigation, Writing – original draft. JM: Investigation, Writing – original draft. MW: Investigation, Writing – original draft. WC: Investigation, Writing – original draft. JiC: Investigation, Supervision, Writing – review & editing. QS: Investigation, Supervision, Writing – review & editing. SX: Conceptualization, Data curation, Formal analysis, Funding acquisition, Investigation, Methodology, Project administration, Resources, Software, Supervision, Validation, Visualization, Writing – original draft, Writing – review & editing.

## Supplementary material

SUPPLEMENTARY FIGURE 1 HA with ORSP modulated the gut microbiota of rats according to the α-diversity and linear discriminant analysis. The Ace **(A)**, Simpson **(B)**, Chao **(C)**, Shannon **(D)**, and Coverage **(E)** indices of α-diversity analysis at the OTU level were calculated and compared **(F)** between two groups. Gut microbiota comparisons from phylum to genus between two groups by linear discriminant analysis effect size (LEfSe) analysis. Linear discriminant analysis (LDA) scores for the differentially abundant bacterial taxa between two groups were calculated by LEfSe.
