# Supplementary figures and images for: Heat acclimation with probiotics-based ORS supplementation alleviates heat stroke-induced multiple organ dysfunction via improving intestinal thermotolerance and modulating gut microbiota in rats
Source: Front Microbiol. 2024 Jun 19;15:1385333. doi: 10.3389/fmicb.2024.1385333 (PMC11220321; doi:10.3389/fmicb.2024.1385333)

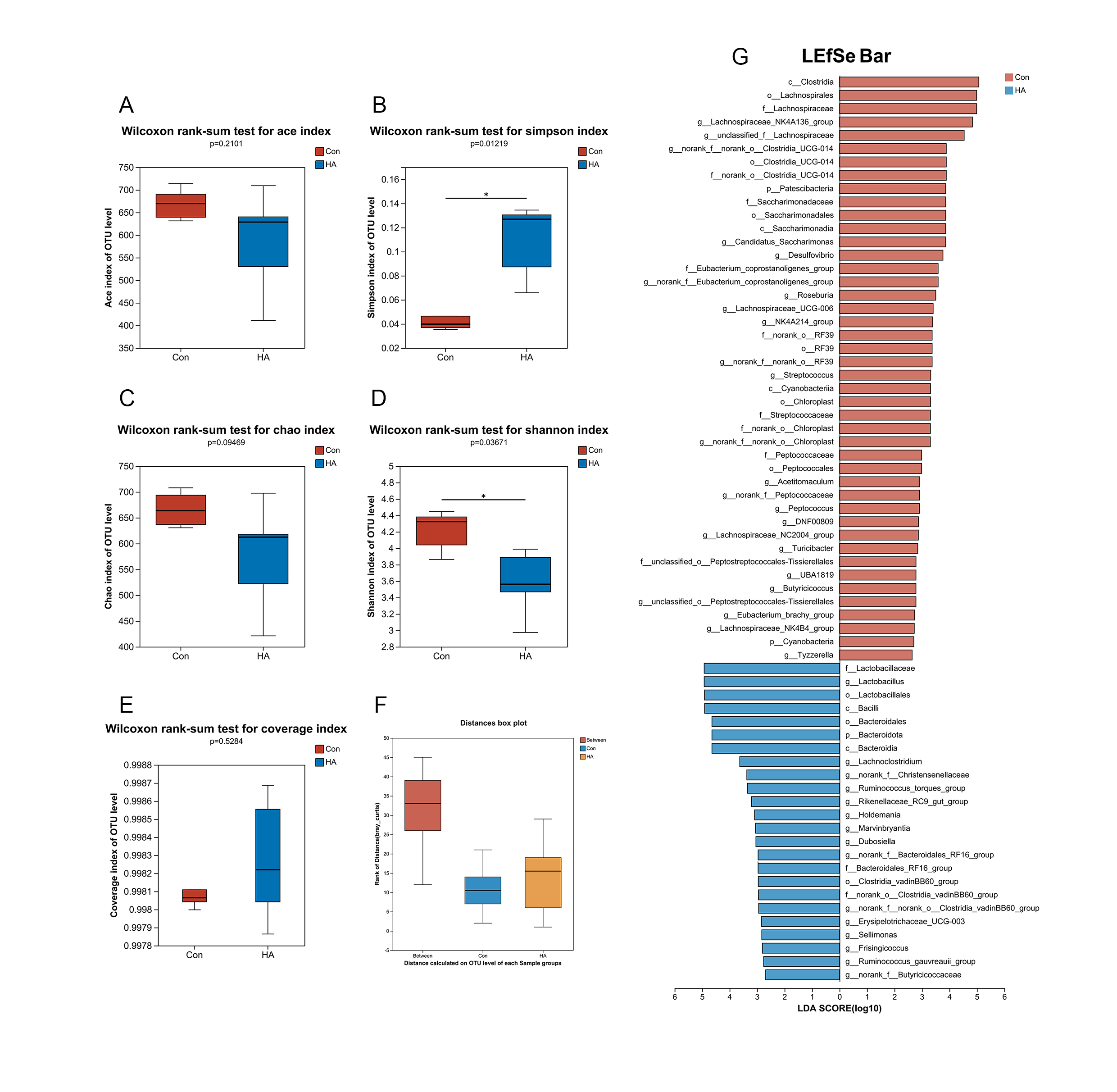

Supplement: SUPPLEMENTARY FIGURE 1 — HA with ORSP modulated the gut microbiota of rats according to the α-diversity and linear discriminant analysis. The Ace (A), Simpson (B), Chao (C), Shannon (D), and Coverage (E) indices of α-diversity analysis at the OTU level were calculated and compared (F) between two groups. Gut microbiota comparisons from phylum to genus between two groups by linear discriminant analysis effect size (LEfSe) analysis. Linear discriminant analysis (LDA) scores for the differentially abundant bacterial taxa between two groups were calculated by LEfSe. [file Image_1.JPEG]

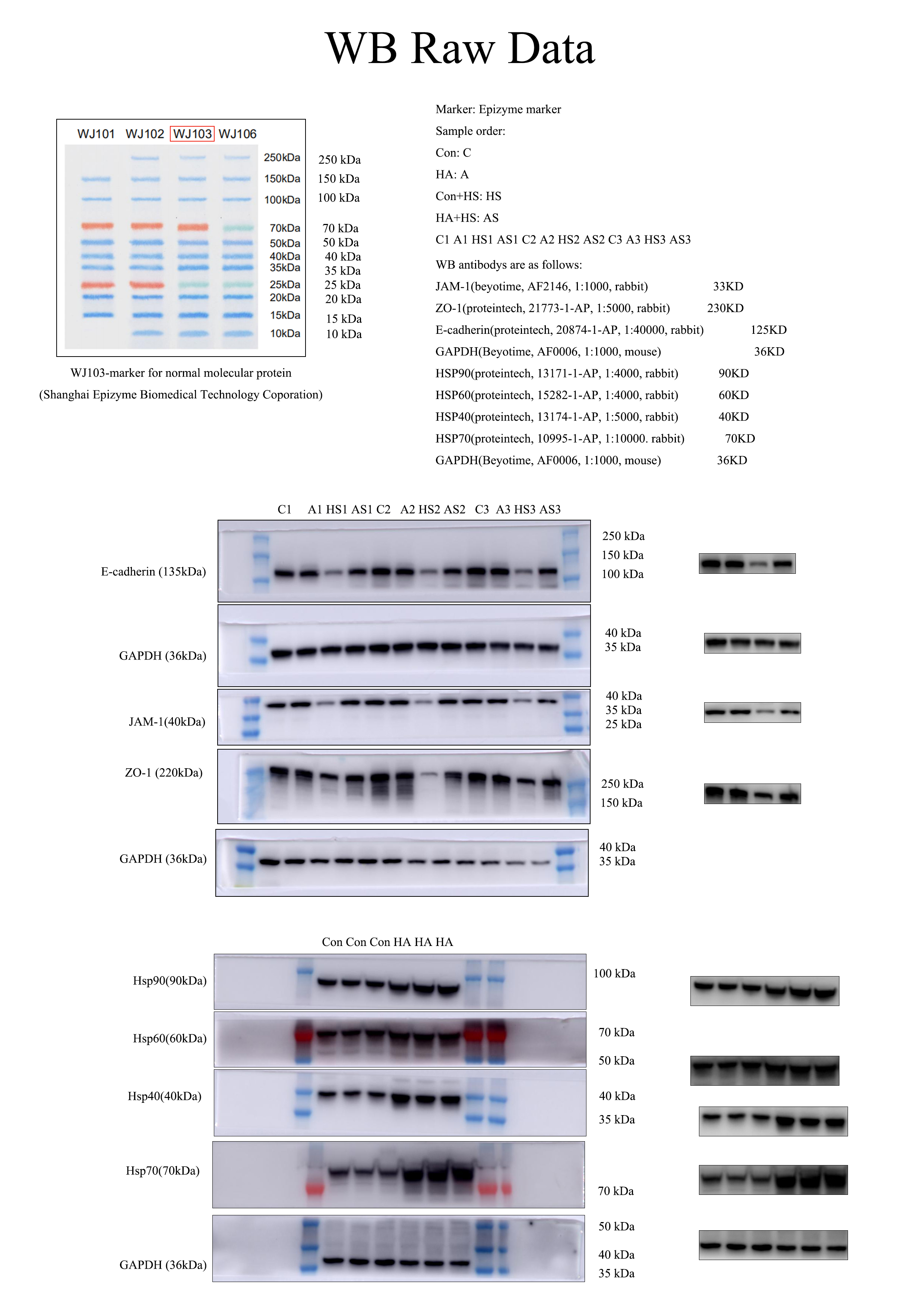

Supplement: Supplementary file 2 [file Data_Sheet_1.ZIP › Figure4 WB RAW DATA.jpg]
